# 90‐Day Repeated‐Dose Toxicological Assessment of MUVZ in Sprague Dawley Rats: Subchronic Study

**DOI:** 10.1155/jt/9540593

**Published:** 2026-10-13

**Authors:** Devanand Shanmugasundaram

**Affiliations:** ^1^ Compliance and Operations (Toxicology) Vedic Lifesciences Pvt. Ltd. Mumbai India

**Keywords:** 90-day repeated dose, MUVZ, OECD Guideline 408, subchronic, *Vitex negundo*, *Zingiber officinale*

## Abstract

**Objectives:**

*Vitex negundo* and *Zingiber officinale* are predominantly used in day‐to‐day life as food or supplements. Both are being reported individually for therapeutic properties. A food supplement consisting of leaves and twigs of *Vitex negundo* and rhizome of *Zingiber officinale*, the first‐of‐a‐kind proprietary blend extract (MUVZ), was developed for joint health.

**Methods:**

As part of the safety profile, a subchronic toxicity study was performed in Sprague Dawley rats under compliance with Good Laboratory Practice (GLP). The study aimed to determine the potential toxicity of MUVZ following oral (gavage) administration by following Organisation for Economic Co‐operation and Development (OECD) Guideline 408. MUVZ was administered for 90 days, with a recovery period of 28 days. MUVZ was administered at 0 (G1), 1000 (G2), 2000 (G3) and 3000 (G4) mg.kg^−1^ body weight (*n* = 10/sex/group); vehicle control (G1R) and high‐dose recovery groups 3000 mg.kg^−1^·b.w (G4R) (*n* = 5/sex/group).

**Results:**

No MUVZ‐related effects were observed in mortality, morbidity, detailed clinical signs, body weight and feed consumption. Statistical significance was found in levels of clinical pathology parameters; however, there was no dose dependency, and further correlative histopathological findings were observed. At the end of the experimental trial, no test article–induced gross and microscopic pathological lesions were observed across the treatment groups.

**Conclusions:**

Based on the findings, no toxicologically significant findings were derived at the highest dose of 3000 mg.kg^−1^ body weight.

## 1. Introduction

*Vitex* *negundo* ‘Nirgundi’ is native to East African, South Asian and American countries [1]; *V*. *negundo* parts (leaf, shoot, root and flower) are used for numerous ailments owing to its anticipated healing properties [2]. *V*. *negundo* has already been explored for anti‐hyperglycaemic, anti‐inflammatory, hypouricemic, analgesic and anti‐itching activity [3]. It is of huge interest in the health sector due to its abundant levels of secondary metabolites, i.e., terpenoids, lignans, flavonoids, alkaloids and glycosides [4].

*Zingiber officinale* ‘Ginger’ is one of the promising spices in the Asian region for medicinal properties and traditional use. The existence of bioactive components in the rhizome, viz., gingerols, terpenes, flavones, flavonoids, quercetin, gingerdiols, paradols, etc., enhances the pharmacological activities [5]. Past clinical trial data suggest ginger improves pain in osteoarthritis, delayed onset muscle soreness (DOMS) and chronic low back pain [6].

Accumulating evidence suggests a high rise in the prevalence of musculoskeletal/joint pain–related concerns in developing countries [7]. Furthermore, in the elderly and female population, these pain conditions are associated with depression and anxiety [8]. Though many pharmacology interventions exist for joint pain, they often end up with side effects and high costs [9]. To overcome this, Enovate Biolife came up with an innovation in developing a proprietary blend of *Vitex negundo* and *Zingiber officinale* for low back support and joint mobility.

For acute effects, reports suggest that the median lethal dose (LD_50_) of *Vitex negundo* was found to be > 5000 mg.kg^−1^ body weight (b.wt) [10], with a no observed adverse effect level (NOAEL) of 1000 mg.kg^−1^ b.wt [11]. For subchronic effects, NOAEL of *Zingiber officinale* in rats was found to be > 1000 mg.kg^−1^ b.wt [12]. U.S. Food and Drug Administration (FDA) established a daily approved intake of up to 4 g in humans [13]. To further expand the database on safety, the existing study was designed to find the safest dose after subchronic oral exposure in rats. These data will then inform us to derive the MUVZ ingredient as Generally Recognised As Safe (GRAS). In Sprague Dawley rats, MUVZ was screened for a 14‐day repeated‐dose range‐finding study and found to be safe at 3000 mg.kg^−1^ b.wt. Based on this outcome, a 90‐day subchronic toxicity study was intended in Sprague Dawley rats to assess the NOAEL of MUVZ as per Organisation for Economic Co‐operation and Development (OECD) Test Guideline 408.

## 2. Materials and Methods

### 2.1. Study Compliance and Animal Welfare

A subchronic toxicity study was performed in compliance with OECD principles of Good Laboratory Practice (GLP) (C(97)186/Final, 26^th^ November 1997) [14] and OECD Test Guideline 408, adopted 25^th^ June 2018 [15]. Experimental protocol was approved by the Institutional Animal Ethics Committee (IAEC) and Committee for the Control and Supervision of Experiments on Animals (CCSEA) (Protocol No. VIP‐IAEC‐356‐2023 dated 30^th^ June 2022) of the Test Facility: Vipragen Biosciences Private Limited, Mysore, where this 90‐day repeated oral toxicity study was conducted. The AAALAC accredited the test facility, and all procedures were executed in compliance with IAEC and CCSEA procedures [16].

### 2.2. Test Article

MUVZ is a proprietary, patent‐pending product of Enovate Biolife Pvt. Ltd. that was prepared by the following cGMP practices. It is a sequential blending of *Zingiber officinale* actives (supercritically extracted under controlled temperature and pressure) with *Vitex negundo* extract (pulverized, extracted using water and spray‐dried). The blend was dried in a vacuum tray dryer (VTD). The final extract was pulverized, sieved and packed. The test article was a yellowish green to brown colour powder with Batch No.: VGBE‐8180002‐22‐23; Manufacture Date: March 2023; and Expiry Date: February 2025. Absence of heavy metals was confirmed using inductively coupled plasma mass spectrometry (ICP‐MS), while microbiological analysis was performed using standard procedures from United States Pharmacopoeia USP 30 NF 25 < 2021>. Negundoside (*Vitex Negundo*) and gingerols (*Zingiber Officinale*) in MUVZ were identified using the HPLC, and total flavonoids were measured by a UV spectrophotometer (refer to supporting information S1–S4).

### 2.3. Experimental Animals and Welfare

Male and female Sprague Dawley rats (6–7 weeks) were obtained from Hylasco Biotechnology, India (Reg. No. 1808/PO/RcBt/S/15/CPCSEA), and housed in a GLP‐accredited facility equipped with an HVAC system. Healthy male and female rats were acclimatized for a minimum of 5 days. A veterinarian verified female rats for nulliparous and nonpregnant status. Rats were maintained in controlled environment conditions with 19.3°C–23.6°C temperature, 47%–69% relative humidity and a 12‐h light/dark cycle [16].

Rats of the same sex/group were housed in polycarbonate rodent cages (size: 412 mm × 290 mm × 190 mm) with a maximum of two per cage, with amenities for food and drinking water bottles. The cage was rotated once a week, and the bedding material was changed once every 2‐3 days or whenever required. Autoclaved pellet feed (Purina Lab Diet, Lot No. 16FEB20232, US) and reverse osmosis–treated water were provided ad libitum. However, during scheduled fasting (overnight), feed was withdrawn from the cages. The corncob bedding material (Rowan Agro Nature Pvt. Ltd, India) was screened for microbial and contaminant testing before the experimental trial [16].

### 2.4. Experimental Design

50 male and 50 female rats were randomised (± 20% mean b.wt) to four main groups (10/sex/group) and two recovery groups (5/sex/group). Study groups are Group 1 (G1): vehicle control (0 mg.kg^−1^ b. wt.); Group 2 (G2): low‐dose MUVZ (1000 mg.kg^−1^ b. wt.); Group 3 (G3): mid‐dose MUVZ (2000 mg.kg^−1^ b. wt.)); Group 4 (G4): high‐dose MUVZ (3000 mg.kg^−1^ b. wt.); Group 1R (G1R): vehicle control recovery (0 mg.kg^−1^ b. wt.); and Group 4R (G4R): high dose of MUVZ recovery (3000 mg.kg^−1^ b. wt.).

Based on the solubility and syringeability test, Milli‐Q water served as a vehicle in G1. Dose formulations (suspension) were freshly prepared at 0, 100, 200 and 300 mg.mL^−1^ and administered orally within 2 h of preparation at a volume of 10 mL.kg^−1^ b. wt. Milli‐Q water and MUVZ were administered as a single daily dose via gavage (14/16/18 gauge size) for 90 days. Recovery groups (G1R and G4R) also followed a similar procedure for 90 days but were kept for a recovery period of 28 days without treatment. A repeated‐dose range‐finding study (14 days) for MUVZ was conducted in SD rats to select the optimum doses for the subchronic study; data were not published.

### 2.5. In‐Life Observations

Mortality and morbidity were scheduled twice daily, and clinical signs were scheduled once daily. Clinical signs are not limited to alterations in eyes, skin, mucus membranes, gait, autonomic activity, posture and response, etc. Measurement of b.wt and feed consumption was documented weekly throughout the experiment. Ocular examination was performed using a 3.5v Coaxial Ophthalmoscope (Welch Allyn Inc., USA) before initiation and during the last week of the experiment in G1 and G4 groups. Before the examination, mydriasis was induced by instilling a 1% solution of tropicamide. A functional observational battery (FOB) was performed in the main and recovery groups during the last week of exposure [16].

### 2.6. Clinical Pathology

At terminal sacrifice, blood samples from all groups (G1–G4, G1R–G4R) were collected from the retro‐orbital plexus in 10% dipotassium ethylenediaminetetraacetic acid (haematology), sodium citrate (coagulation) and plain tubes (clinical chemistry) [16].

Haematology parameters (i.e., red blood cell count [RBC], total leukocyte count [WBC], haemoglobin [HGB], platelet count [PLT], haematocrit [HCT], etc.) were examined using a Siemens ADVIA 2120i Haematology Analyser. Prothrombin time (PT) and activated partial thromboplastin time (APTT) were analysed by Diagnostica Stago, 92600 Asnieres, France [17]. Clinical chemistry parameters (i.e., total protein [T.Pro], glucose [GLU], albumin [ALB], alanine aminotransferase [ALT], aspartate aminotransferase [AST], high‐density lipoprotein [HDL], low‐density lipoprotein [LDL], total cholesterol [TCHO], creatinine [CRE], etc.) were analysed using Randox Daytona Plus, London, UK [18]. Calcium (Ca), phosphorus (I.Phos), chloride (Cl^−^), sodium (Na^+^) and potassium (K^+^) were analysed using the 9180 Electrolyte Analyser, Roche, USA [18]. Urinalysis parameters (i.e., appearance/clarity, colour, specific gravity, etc.) were analysed using a Cobas u411 Urine Analyser (Roche, USA) [18, 19]. Serum thyroid hormones, viz., triiodothyronine (T3), thyroxine (T4) and thyroid‐stimulating hormone (TSH), were analysed using Robonik India by the ELISA method (kits from Wuhan Huamei Biotech Co., Ltd., P.R. China) [16, 20].

### 2.7. Oestrus Cycle

On the day of the scheduled necropsy, the oestrus cycle was determined in all female rats. The assessment was conducted in accordance with the laboratory’s established SOP using the vaginal lavage (flush) method, as outlined below:

A small volume of sterile saline/Milli‐Q water was gently flushed into the vaginal canal of the animal. The recovered vaginal wash was transferred onto a clean glass slide. The slide was then examined microscopically to determine the corresponding stage of the oestrous cycle.

### 2.8. Pathology and Histopathology (HP)

At terminal sacrifice, animals were euthanized by CO_2_ asphyxiation method on Day 91 for the main groups and Day 119 for the recovery groups. A complete gross examination was performed, including an external body surface and cranial, thoracic and abdominal cavities, as well as a detailed internal visceral examination.

Organs, i.e., bone marrow (femur), colon, duodenum, epididymides, oesophagus, heart, kidneys, liver, ovaries, pancreas, pituitary gland, prostate + seminal vesicles with coagulating glands, spleen, thymus, thyroid with parathyroids, trachea, urinary bladder, uterus with cervix, vagina, etc., were collected, weighed and fixed in 10% buffer neutral formalin. Eyes and testes were preserved in modified Davidson’s fluid. Fixed tissues were processed using the routine paraffin embedding technique and stained with Mayer’s haematoxylin and eosin (H&E) stain. In contrast, testes were stained with periodic acid–Schiff (PAS) and haematoxylin for qualitative assessment of spermatogenesis. HP examination was conducted on either the sexes of G1 and G4 preserved organs/tissues [16].

### 2.9. Statistical Analysis

b.wt, feed pattern, clinical pathology, FOB and absolute and relative organ weights were checked for statistical analyses using GraphPad Prism Version 10.0.2. Statistical analyses were performed separately for male and female animals. Treatment groups within each sex were compared with their respective concurrent control group using one‐way analysis of variance (ANOVA) followed by an appropriate multiple comparison test. No direct statistical comparisons between male and female animals were performed. Data are presented as mean ± standard deviation (SD), and statistical significance was considered at *p* ≤ 0.05 [21].

## 3. Results

### 3.1. Mortality and Clinical Signs

MUVZ‐related morbidity and mortality were not found in G1–G4 and G1R–G4R groups. No adverse clinical signs were observed during the study. However, mild salivation was observed in G2 (from Days 41 and 43 in males and females), G3 (from Days 13 and 12 in males and females), G4 (from Day 2 and Day 3 in males and females) and G4R (from Day 3 in males and females). In all groups, salivation was transient, disappeared after 1 h of post‐dose observation and hence was not considered an adverse effect of MUVZ.

### 3.2. Ophthalmological and FOB Observation

No ocular alterations were observed in the main and recovery groups during the initial or terminal exposure.

No MUVZ‐related FOB deficits were observed in G2–G4 and G4R animals. Nevertheless, a statistically significant change in foot splay (↓) was observed in G2–G4 when compared with G1 in males. Decrease in foot play was not MUVZ related, as it is observed in single‐sex and could not be corroborated with any other clinical signs.

### 3.3. b.wt and Feed Consumption

No MUVZ‐related b.wt changes were observed in males and females across the main and recovery groups (Table 1). However, in b.wt gain, a statistically significant change (↓) was observed in G4R males (Days 43–50 and 85–92) and G4R females (Days 15–22) when compared with G1R.

**TABLE 1:** Initial and terminal body weight (g) following repeated oral administration of MUVZ to Sprague Dawley rats for 90 days.

Group		Bodyweight (g)			
		Male		Female	
		Initial week	Terminal week	Initial week	Terminal week
G1	Vehicle control	256.24 ± 8.25	620 ± 33.36	191.43 ± 7.48	314.88 ± 19.71
G2	MUVZ 1000 mg.kg^−1^ b.wt	254.57 ± 8.54	615 ± 31.06	192.12 ± 7.04	316.37 ± 15.18
G3	MUVZ 2000 mg.kg^−1^ b.wt	255.75 ± 7.28	618 ± 40.92	192.74 ± 9.08	322.82 ± 19.93
G4	MUVZ 3000 mg.kg^−1^ b.wt	255.74 ± 7.26	621 ± 52.72	192.16 ± 12.07	318.41 ± 22.49
G1R	Recovery control (vehicle)	278.44 ± 1.88	673 ± 42.80	207.15 ± 4.32	360.63 ± 8.50
G4R	Recovery MUVZ 3000 mg.kg^−1^ b.wt	279.01 ± 3.84	678 ± 46.90	207.91 ± 3.28	363.54 ± 13.34

*Note:* Values are presented as mean ± SD (main groups, *n* = 10 each, and recovery groups, *n* = 5 each). Keys: mg, milligram; kg: kilogram; b.wt, body weight.

Abbreviations: g: gram; G: group; R: recovery.

No MUVZ‐treated changes in food intake patterns were observed in males and females of G1–G4 and G1R–G4R groups. In G2 females, a statistically significant change (↑) was observed during Days 22–29, in comparison with the G1 group. Statistical changes in b.wt gain and feed consumption are confined to single sex and are not dose‐dependent.

### 3.4. Clinical Pathology

#### 3.4.1. Haematology

No MUVZ‐related biological changes were observed in haematology parameters of G2–G4 and G4R. However, a statistically significant change in absolute neutrophil count (10^3^/μL) (↑) was observed in G2 when compared to the G1 males. In females, statistically significant changes were observed for MPV (↓) and reticulocyte (↑) at G2; HGB (↓) at G3; neutrophil (10^3^/μL) (↑) at G4; and relative monocyte count (↑) at G4R when compared to respective control groups. In females, PT (↑) was observed in G4 when compared to G1 (Table 2).

**TABLE 2:** Haematological parameters following repeated oral administration of MUVZ to Sprague Dawley rats for 90 days.

Parameters	Sex	Group					
		G1	G2	G3	G4	G1R	G4R
		Vehicle control	MUVZ	MUVZ	MUVZ	Recovery control (vehicle)	Recovery MUVZ
			1000 mg.kg^−1^ b.wt.	2000 mg.kg^−1^ b.wt.	3000 mg.kg^−1^ b.wt.		3000 mg.kg^−1^ b.wt.
RBC (10^6^/μL)	Male	8.69 ± 0.21	8.79 ± 0.31	8.90 ± 0.38	8.76 ± 0.37	8.70 ± 0.69	8.70 ± 0.80
	Female	8.01 ± 0.24	7.61 ± 0.47	7.50 ± 0.82	7.74 ± 0.68	8.20 ± 0.47	7.89 ± 0.21

HGB (g/dL)	Male	16.15 ± 0.42	16.09 ± 0.47	16.45 ± 0.48	15.80 ± 0.43	16.06 ± 0.58	16.44 ± 0.70
	Female	15.90 ± 0.35	15.34 ± 0.66	14.75 ± 1.41^∗^	15.15 ± 0.90	15.84 ± 0.35	15.54 ± 0.52

HCT (%)	Male	47.59 ± 1.31	47.92 ± 1.41	48.90 ± 1.47	46.90 ± 1.65	46.08 ± 1.44	47.42 ± 2.28
	Female	45.95 ± 1.57	44.06 ± 1.73	43.00 ± 4.25	44.52 ± 2.95	45.40 ± 1.16	44.80 ± 1.39

PLT (10^3^/μL)	Male	989.90 ± 81.78	1052.90 ± 185.80	883.60 ± 215.04	1014.90 ± 168.81	1154.80 ± 67.71	1094.80 ± 157.47
	Female	980.70 ± 66.98	1048.60 ± 104.12	966.40 ± 154.49	1072.50 ± 124.01	1125.00 ± 113.85	942.80 ± 233.22

MCV (fL)	Male	54.79 ± 1.82	54.53 ± 1.19	54.99 ± 1.79	53.56 ± 1.02	53.18 ± 3.13	54.66 ± 2.60
	Female	57.40 ± 1.83	58.06 ± 2.46	57.39 ± 1.13	57.63 ± 1.91	55.52 ± 3.17	56.80 ± 0.89

MCH (pg)	Male	18.60 ± 0.60	18.31 ± 0.52	18.49 ± 0.74	18.04 ± 0.35	18.52 ± 1.11	18.94 ± 1.08
	Female	19.86 ± 0.53	20.24 ± 0.70	19.69 ± 0.47	19.62 ± 0.80	19.36 ± 1.11	19.70 ± 0.35

MCHC (g/dL)	Male	33.94 ± 0.31	33.58 ± 0.51	33.63 ± 0.51	33.71 ± 0.50	34.84 ± 0.31	34.64 ± 0.40
	Female	34.60 ± 0.70	34.82 ± 0.33	34.34 ± 0.49	34.05 ± 0.62	34.86 ± 0.43	34.66 ± 0.17

RDW (%)	Male	13.90 ± 0.43	14.14 ± 0.66	14.00 ± 0.52	14.08 ± 0.36	14.08 ± 0.81	13.18 ± 0.70
	Female	12.76 ± 0.34	12.89 ± 0.91	12.75 ± 0.40	13.21 ± 0.94	13.66 ± 1.05	12.70 ± 0.16

MPV (fL)	Male	9.91 ± 0.42	9.85 ± 0.51	10.13 ± 0.75	9.97 ± 0.26	9.74 ± 0.35	9.68 ± 0.72
	Female	10.20 ± 0.48	9.54 ± 0.39^∗^	9.94 ± 0.37	9.86 ± 0.57	10.18 ± 0.44	10.38 ± 1.06

Retic (%)	Male	2.36 ± 0.19	2.21 ± 0.20	2.30 ± 0.19	2.50 ± 0.16	2.38 ± 0.23	2.42 ± 0.28
	Female	2.33 ± 0.15	2.59 ± 0.28^∗^	2.51 ± 0.23	2.57 ± 0.21	2.48 ± 0.11	2.46 ± 0.15

WBC (10^3^/μL)	Male	9.82 ± 1.08	11.37 ± 2.91	10.55 ± 1.79	10.23 ± 2.34	10.64 ± 1.04	8.93 ± 2.54
	Female	7.73 ± 1.98	7.21 ± 1.35	7.77 ± 2.08	8.88 ± 2.37	8.41 ± 2.46	6.02 ± 0.78

NEU (%)	Male	19.79 ± 3.34	23.30 ± 4.99	20.22 ± 5.50	24.06 ± 9.83	25.88 ± 4.71	22.04 ± 4.35
	Female	15.40 ± 5.68	17.42 ± 4.51	19.18 ± 6.09	18.86 ± 3.55	23.84 ± 4.93	20.10 ± 3.18

LYM (%)	Male	71.77 ± 4.37	68.87 ± 6.33	70.36 ± 6.62	67.55 ± 11.55	66.44 ± 4.21	69.02 ± 5.73
	Female	77.94 ± 6.96	75.90 ± 5.15	74.24 ± 6.96	73.76 ± 3.55	68.52 ± 5.31	70.222.26

MON (%)	Male	6.37 ± 1.36	5.93 ± 1.33	7.22 ± 1.45	6.42 ± 2.67	5.20 ± 0.72	6.08 ± 1.52
	Female	4.71 ± 1.18	4.36 ± 0.94	4.43 ± 0.92	5.19 ± 1.04	5.00 ± 0.94	6.42 ± 0.76^∗^

EOS (%)	Male	2.07 ± 0.72	1.90 ± 0.62	2.20 ± 0.79	1.97 ± 0.44	2.48 ± 0.22	2.86 ± 0.88
	Female	1.95 ± 0.62	2.32 ± 0.48	2.15 ± 0.58	2.19 ± 0.60	2.64 ± 0.53	3.26 ± 0.57

BAS (%)	Male	0.00 ± 0.00	0.00 ± 0.00	0.00 ± 0.00	0.00 ± 0.00	0.00 ± 0.00	0.00 ± 0.00
	Female	0.00 ± 0.00	0.00 ± 0.00	0.00 ± 0.00	0.00 ± 0.00	0.00 ± 0.00	0.00 ± 0.00

PT (seconds)	Male	10.34 ± 0.73	11.32 ± 1.78	10.69 ± 1.11	10.90 ± 1.14	14.30 ± 2.24	12.14 ± 1.53
	Female	10.62 ± 1.37	11.24 ± 1.70	10.89 ± 0.74	13.36 ± 3.04^∗^	12.20 ± 1.97	12.76 ± 1.63

APTT (seconds)	Male	15.38 ± 1.03	16.14 ± 2.05	16.42 ± 2.11	15.01 ± 1.24	16.24 ± 1.66	18.66 ± 1.95
	Female	17.94 ± 3.86	16.36 ± 2.04	15.72 ± 2.13	16.37 ± 1.93	18.92 ± 0.95	18.78 ± 2.51

NEU (10^3^/μL)	Male	1.94 ± 0.36	2.66 ± 0.91^∗^	2.10 ± 0.61	2.29 ± 0.63	2.74 ± 0.51	2.05 ± 0.91
	Female	1.17 ± 0.44	1.25 ± 0.40	1.44 ± 0.43	1.65 ± 0.46^∗^	2.06 ± 0.84	1.20 ± 0.10

*Not*e: Values are presented as mean ± SD (main groups treated for 90 days, *n* = 10 each; recovery groups, *n* = 5). Keys: mg, milligram; kg: kilogram; b. wt, body weight; μL, microlitre; dL, decilitre; fL, femtolitres; pg, picograms; %, percentage; RBC, red blood cell count; HGB, haemoglobin; HCT, haematocrit; PLT, platelet count; RDW, red cell distribution width; Retic, reticulocyte count; WBC, white blood cell count; NEU, neutrophils; LYM, lymphocytes; MON, monocytes; EOS, eosinophils; BAS, hbasophils.

Abbreviations: APTT, activated partial thromboplastin time; g, gram; G, group; R, recovery; MCV, mean corpuscular volume; MCH, mean corpuscular haemoglobin; MCHC, mean corpuscular haemoglobin concentration; MPV, mean platelet volume; PT, prothrombin time.

^∗^Statistically significant from the vehicle control group (*p* ≤ 0.05).

#### 3.4.2. Clinical Chemistry

No MUVZ‐related biological changes were observed in the clinical chemistry parameters of G2–G4 and G4R. Statistically significant changes were observed in males for calcium (↓) and total bilirubin (↑) in G2; CRE (↑) and TCHO (↓) in G3; and CRE (↑) and total bilirubin (↑) in G4, when compared to G1. Also, in G4R, statistically significant changes were observed for AST (↓), calcium (↓), potassium (↓) and chloride (↑) when compared to G1R. Compared to G1, statistical significances were limited to a decrease in urea (↓), CRE (↓), BUN (↓) and sodium (↓) in G2 and an increase in TCHO (↑), HDL (↑) and sodium (↑) in G4 of females. Also, in G4R, statistically significant changes were observed for GLU (↑) when compared to G1R (Table 3). Observed statistical changes in clinical chemistry lacked dose dependency and were not correlated with gross and HP findings.

**TABLE 3:** Clinical chemistry parameters following repeated oral administration of MUVZ to Sprague Dawley rats for 90 days.

Parameters	Sex	Group					
		G1	G2	G3	G4	G1R	G4R
		Vehicle control	MUVZ	MUVZ	MUVZ	Recovery control (vehicle)	MUVZ
			1000 mg.kg^−1^ b.wt.	2000 mg.kg^−1^ b.wt.	3000 mg.kg^−1^ b.wt.		3000 mg.kg^−1^ b.wt.
GLU (mg/dL)	Male	116.56 ± 18.93	111.79 ± 18.90	114.56 ± 19.57	105.55 ± 11.71	123.27 ± 16.78	128.45 ± 8.69
	Female	102.98 ± 18.50	96.26 ± 10.95	97.71 ± 14.83	94.16 ± 17.43	112.06 ± 8.96	135.21 ± 17.82^∗^

UREA (mg/dL)	Male	25.99 ± 2.69	27.68 ± 2.63	27.60 ± 2.47	27.76 ± 2.55	26.88 ± 1.62	29.43 ± 3.08
	Female	36.81 ± 6.85	30.36 ± 4.91^∗^	32.34 ± 5.51	31.46 ± 3.03	32.44 ± 2.61	36.15 ± 6.40

CRE (mg/dL)	Male	0.53 ± 0.02	0.57 ± 0.04	0.60 ± 0.06^∗^	0.58 ± 0.03^∗^	0.51 ± 0.03	0.50 ± 0.03
	Female	0.66 ± 0.10	0.55 ± 0.04^∗^	0.57 ± 0.04	0.58 ± 0.08	0.61 ± 0.03	0.63 ± 0.06

TCHO (mg/dL)	Male	49.67 ± 8.08	45.20 ± 8.02	39.53 ± 6.67^∗^	47.08 ± 11.68	76.51 ± 18.30	75.41 ± 12.67
	Female	64.46 ± 14.43	65.17 ± 11.51	73.31 ± 15.24	82.90 ± 16.30^∗^	109.58 ± 19.07	105.95 ± 15.65

TRIG (mg/dL)	Male	55.28 ± 19.44	53.93 ± 10.79	59.02 ± 12.24	55.00 ± 8.68	116.80 ± 35.57	127.91 ± 40.17
	Female	47.83 ± 8.87	42.80 ± 8.27	46.96 ± 7.75	41.60 ± 5.55	67.30 ± 12.62	70.95 ± 12.58

AST (U/L)	Male	126.43 ± 15.85	140.05 ± 27.74	137.80 ± 30.95	129.53 ± 33.53	165.86 ± 20.29	136.70 ± 17.20^∗^
	Female	149.21 ± 74.45	121.99 ± 24.45	111.03 ± 21.03	116.92 ± 24.37	130.63 ± 67.59	152.16 ± 43.97

ALT (U/L)	Male	51.53 ± 11.24	45.67 ± 4.65	59.10 ± 21.40	45.94 ± 6.43	51.45 ± 15.46	41.37 ± 2.02
	Female	56.45 ± 39.16	40.34 ± 7.28	40.95 ± 9.67	36.38 ± 5.10	36.80 ± 10.20	58.14 ± 31.75

TP (g/L)	Male	62.87 ± 2.19	62.84 ± 2.57	63.13 ± 2.32	63.43 ± 2.94	71.86 ± 1.98	72.96 ± 1.64
	Female	77.87 ± 3.94	77.36 ± 3.49	79.03 ± 4.28	80.80 ± 4.45	82.01 ± 2.89	85.39 ± 2.68

HDL (mg/dL)	Male	24.46 ± 3.49	21.98 ± 2.98	22.48 ± 3.13	26.16 ± 4.07	17.88 ± 6.44	13.32 ± 1.82
	Female	35.51 ± 5.29	36.69 ± 4.54	40.28 ± 6.75	44.27 ± 6.15^∗^	28.27 ± 4.13	27.32 ± 3.87

LDL (mg/dL)	Male	8.30 ± 1.49	9.03 ± 3.59	7.23 ± 1.55	7.85 ± 1.17	5.71 ± 3.03	5.35 ± 0.35
	Female	7.02 ± 1.09	6.28 ± 1.12	7.04 ± 1.62	7.28 ± 1.38	5.74 ± 2.00	4.68 ± 0.79

ALB (g/L)	Male	37.13 ± 0.78	36.83 ± 1.30	37.24 ± 1.26	38.04 ± 1.14	38.17 ± 1.55	38.41 ± 0.77
	Female	45.13 ± 2.52	45.78 ± 2.49	46.37 ± 2.69	47.60 ± 3.45	44.55 ± 2.55	47.60 ± 2.10

T.BIL (mg/dL)	Male	0.17 ± 0.04	0.24 ± 0.07^∗^	0.20 ± 0.05	0.23 ± 0.04^∗^	0.17 ± 0.05	0.11 ± 0.04
	Female	0.23 ± 0.04	0.25 ± 0.05	0.25 ± 0.04	0.22 ± 0.03	0.17 ± 0.05	0.17 ± 0.02

Ca (mg/dL)	Male	10.42 ± 0.53	9.89 ± 0.29^∗^	10.32 ± 0.49	10.14 ± 0.23	10.55 ± 0.51	9.93 ± 0.16^∗^
	Female	11.19 ± 0.42	11.05 ± 0.66	11.28 ± 0.27	11.34 ± 0.51	10.49 ± 0.28	10.74 ± 0.20

PHO (mg/dL)	Male	7.30 ± 0.35	7.47 ± 0.72	7.75 ± 0.61	7.70 ± 0.45	6.64 ± 0.43	6.07 ± 0.43
	Female	6.83 ± 1.58	5.77 ± 0.74	5.91 ± 1.08	6.36 ± 2.11	4.65 ± 0.69	4.52 ± 1.13

GLOB (g/L)	Male	25.75 ± 1.64	26.01 ± 1.54	25.89 ± 1.48	25.39 ± 1.98	33.69 ± 1.19	34.54 ± 1.63
	Female	32.74 ± 1.57	31.59 ± 1.84	32.66 ± 1.90	33.20 ± 1.66	37.47 ± 1.74	37.80 ± 1.26

TBA (μmol/L)	Male	25.34 ± 30.20	41.05 ± 41.04	57.96 ± 62.03	18.04 ± 13.25	13.51 ± 9.43	14.14 ± 12.55
	Female	68.76 ± 45.06	58.56 ± 45.59	39.55 ± 50.88	49.56 ± 47.76	33.35 ± 16.72	14.24 ± 11.72

ALP (U/L)	Male	64.79 ± 20.68	63.77 ± 12.47	82.19 ± 22.50	70.90 ± 16.63	106.12 ± 32.20	97.90 ± 28.93
	Female	46.08 ± 12.63	47.76 ± 19.95	40.25 ± 11.50	42.18 ± 10.16	46.07 ± 9.38	39.10 ± 10.38

BUN (mg/dL)	Male	12.14 ± 1.26	12.93 ± 1.23	12.90 ± 1.15	12.97 ± 1.19	12.56 ± 0.76	13.75 ± 1.44
	Female	17.20 ± 3.20	14.19 ± 2.29^∗^	15.11 ± 2.57	14.70 ± 1.42	15.16 ± 1.22	16.89 ± 2.99

*A*/*G* ratio	Male	1.45 ± 0.08	1.42 ± 0.06	1.44 ± 0.07	1.50 ± 0.09	1.13 ± 0.06	1.11 ± 0.06
	Female	1.38 ± 0.04	1.45 ± 0.10	1.42 ± 0.06	1.43 ± 0.09	1.19 ± 0.09	1.26 ± 0.06

Na (mmol/L)	Male	140.70 ± 0.67	140.40 ± 1.07	140.50 ± 1.18	140.10 ± 0.99	142.00 ± 0.71	141.80 ± 0.45
	Female	143.10 ± 2.33	141.10 ± 1.10^∗^	143.30 ± 1.77	145.10 ± 1.52^∗^	141.60 ± 0.55	140.80 ± 0.45

K (mmol/L)	Male	5.02 ± 0.35	5.16 ± 0.39	5.22 ± 0.41	5.30 ± 0.32	5.02 ± 0.19	4.52 ± 0.29^∗^
	Female	4.85 ± 0.34	4.93 ± 0.40	5.10 ± 0.52	4.93 ± 0.16	4.96 ± 0.17	4.74 ± 0.48

Cl (mmol/L)	Male	102.90 ± 0.74	102.10 ± 1.45	102.50 ± 1.35	101.90 ± 0.88	103.00 ± 0.00	104.20 ± 0.84^∗^
	Female	103.50 ± 1.96	102.50 ± 1.58	104.00 ± 1.56	105.10 ± 0.74	103.40 ± 0.89	102.80 ± 1.79

T3 (ng.mL^−1^)	Male	0.969 ± 0.022	1.008 ± 0.025	0.976 ± 0.013	1.008 ± 0.023^∗^	0.956 ± 0.038	0.895 ± 0.024^∗^
	Female	0.991 ± 0.033	1.023 ± 0.025	1.000 ± 0.032	1.036 ± 0.029^∗^	0.968 ± 0.024	0.934 ± 0.035

T4 (ng.mL^−1^)	Male	40.528 ± 0.410	40.616 ± 0.337	40.382 ± 0.234	40.302 ± 0.320	40.546 ± 0.499	40.989 ± 0.365
	Female	41.087 ± 0.444	41.270 ± 0.276	41.285 ± 0.289	41.051 ± 0.358	41.154 ± 0.548	40.774 ± 0.266

TSH (ng.mL^−1^)	Male	1411.069 ± 193.711	1738.976 ± 224.528	2019.682 ± 144.304^∗^	1950.472 ± 96.526^∗^	1606.904 ± 112.118	1604.190 ± 38.705^∗^
	Female	1474.136 ± 151.024	1624.410 ± 303.539	1686.384 ± 93.551^∗^	1468.379 ± 209.653^∗^	1707.274 ± 101.615	1579.052 ± 67.623^∗^

*Note:* Values are presented as mean ± SD (main groups treated for 90 days, *n* = 10 each; recovery groups, *n* = 5). Keys: GLU, glucose; CRE, creatinine; TCHO, total cholesterol; TRIG, triglycerides; AST, aspartate aminotransferase; ALT, alanine aminotransferase; HDL, high‐density lipoprotein cholesterol; LDL, low‐density lipoprotein cholesterol; ALB, albumin; T.BIL, total bilirubin; Ca, calcium; PHO, phosphorous; GLOB, globulin; ALP, alkaline phosphatase; Na, sodium; K, potassium; Cl, chloride; T3, triiodothyronine; T4, thyroxine; mg, milligram; kg, kilograms; b.wt, body weight; dL, decilitre; ng, nanograms; mL, millilitre; μmol, micromoles; mmol, millimoles.

Abbreviations: A/*G* ratio, albumin/globulin ratio; BUN, blood urea nitrogen; g, grams; G, group; L, litres; R, recovery; TP, total protein; TBA, total bile acids; TSH, thyroid‐stimulating hormone; U, units.

^∗^Statistically significant change at *p* < 0.05 in comparison with the respective vehicle control and recovery control vehicle group.

#### 3.4.3. Thyroid Estimation and Urinalysis

Statistically significant changes were observed in TSH levels (↑) of G3 and G4 in comparison with G1 of males and females. Also, decreased TSH levels (↓) were observed in both male and female animals in G4R compared with G1R.

Significant statistical changes were observed for T3 levels (↑) in G4 (male and female) and T3 levels (↓) in G4R males when compared with respective control groups. No statistically significant changes were observed in T4 levels in either sex (Table 3). Observed statistically significant changes were considered sporadic and incidental. Observed T3 and TSH statistical changes were not correlated with absolute thyroid or relative weight and histopathological examinations.

No treatment‐related changes were found in urinalysis parameters across G1–G4 and G1R–G4R groups (Table 4).

**TABLE 4:** Urine parameters following repeated oral administration of MUVZ to Sprague Dawley rats for 90 days.

Parameters		Sex	Groups					
			G1	G2	G3	G4	G1R	G4R
			G1 (0)	MUVZ	MUVZ	MUVZ	Recovery control (vehicle)	Recovery MUVZ
				1000 mg.kg^−1^ b.wt.	2000 mg.kg^−1^ b.wt.	3000 mg.kg^−1^ b.wt.		3000 mg.kg^−1^ b.wt.
Volume (mL)	Mean	Male	13.50 ± 5.30	14.00 ± 5.68	13.50 ± 4.74	13.50 ± 4.74	10.00 ± 5.00	11.00 ± 4.18
		Female	12.00 ± 4.83	11.50 ± 4.12	12.50 ± 3.54	13.50 ± 5.80	10.00 ± 5.00	9.00 ± 4.18

Appearance	Clear	Male	8	8	7	6	4	5
		Female	8	6	7	7	3	3
	ST	Male	2	1	3	4	1	0
		Female	2	4	3	3	2	2
	Turbid	Male	0	1	0	0	—	—
		Female	—	—	—	—	—	—

Specific gravity	Mean	Male	1.02 ± 0.00	1.02 ± 0.00	1.02 ± 0.00	1.02 ± 0.00	1.02 ± 0.01	1.02 ± 0.46
		Female	1.01 ± 0.00	1.01 ± 0.00	1.01 ± 0.00	1.01 ± 0.00	1.01 ± 0.00	1.02 ± 0.01

pH	Mean	Male	5.90 ± 1.10	5.90 ± 0.74	6.30 ± 1.06	6.60 ± 0.52	6.80 ± 1.30	7.40 ± 0.89
		Female	7.40 ± 0.81	7.90 ± 0.32	7.60 ± 0.52	7.50 ± 0.71	7.40 ± 0.55	7.60 ± 0.55

WBC	Neg	Male	7	6	7	2	3	2
		Female	9	6	0	2	1	0
	25	Male	1	2	0	4	2	1
		Female	1	4	8	7	3	2
	100	Male	0	0	0	0	0	2
		Female	0	0	2	1	1	3
	500	Male	2	2	3	4	0	0
		Female	0	0	0	0	0	0

Protein (mg/dL)	Neg	Male	8	8	7	6	5	2
		Female	8	5	8	6	2	2
	25	Male	2	1	2	4	0	3
		Female	2	5	2	4	3	3
	75	Male	0	1	1	0	0	0
		Female	0	0	0	0	0	0

Glucose (mg/dL)	Norm	Male	10	10	10	10	5	5
		Female	10	10	10	10	5	5

Erythrocytes	Neg	Male	10	10	10	10	5	5
		Female	10	8	8	6	3	4
	10	Female	0	1	0	4	0	0
	25		0	1	1	0	2	1
	50		0	0	1	0	0	0

Nitrite	Neg	Male	8	6	8	5	5	4
		Female	10	8	9	6	3	2
	Pos	Male	2	4	2	5	0	1
		Female	0	2	1	4	2	3

Ketone bodies (mg/dL)	Neg	Male	8	8	7	6	4	4
		Female	10	10	9	8	3	3
	5	Male	0	0	0	1	1	1
		Female	0	0	1	2	2	2
	15	Male	2	2	3	3	0	0
		Female	0	0	0	0	0	0

Urobilinogen	Normal	Male	10	10	10	10	5	5
		Female	10	10	10	10	5	5

Bilirubin (mg/dL)	Neg	Male	10	10	10	10	5	5
		Female	10	10	10	10	5	5

Colour	Pale Yellow	Male	10	9	9	9	0	0
		Female	8	7	7	5	5	4
	Yellow	Male	0	1	0	0	5	3
		Female	1	1	2	2	0	0
	Amber	Male	0	0	1	1	0	2
		Female	1	2	1	3	0	1

*Note:* Values are presented as mean ± SD for main (*n* = 10) treated for 90 days and recovery (*n* = 5) groups. Keys: mL, millilitres; mg, milligrams; kg, kilogram; b.wt, body weight; dL, decilitres; WBC, white blood cell count; Neg, negative; Pos, positive.

Abbreviations: G, group; R, recovery; ST, slightly turbid.

### 3.5. Gross Pathology

No MUVZ‐related gross lesions were evident in any of the male or female rats across all treatment groups.

### 3.6. Organ Weights

In male rats, statistically significant decreases in absolute and relative kidney weight **(↓)** were observed in G2 and G3; in G4R, absolute thymus weight **(↓)**, relative spleen **(↓)** and relative thymus weight **(↓)** were observed. In females, a statistically significant absolute and relative liver weight (↑) was observed in G4; absolute (↑) and relative pituitary gland weight (↑) were observed in G4R (Tables 5 and 6).

**TABLE 5:** Absolute organ weight (g) following repeated oral administration of MUVZ to Sprague Dawley rats for 90 days.

Organ	Sex	Group					
		G1	G2	G3	G4	G1R	G4R
		Vehicle control	MUVZ	MUVZ	MUVZ	Recovery control (vehicle)	Recovery MUVZ
			1000 mg.kg^−1^ b.wt.	2000 mg.kg^−1^ b.wt.	3000 mg.kg^−1^ b.wt.		3000 mg.kg^−1^ b.wt.
Liver	Male	20.621 ± 2.920	20.796 ± 3.524	20.856 ± 2.678	21.824 ± 3.188	21.105 ± 2.264	22.384 ± 2.724
	Female	10.446 ± 1.041	11.197 ± 1.512	10.927 ± 1.269	12.262 ± 2.122^∗^	10.156 ± 0.824	12.237 ± 1.138

Kidneys	Male	4.964 ± 0.473	4.287 ± 0.513^∗^	4.382 ± 0.174^∗^	4.543 ± 0.600	4.888 ± 0.550	4.865 ± 0.188
	Female	2.273 ± 0.129	2.364 ± 0.272	2.228 ± 0.228	2.529 ± 0.289	2.443 ± 0.166	2.545 ± 0.224

Spleen	Male	1.043 ± 0.151	1.010 ± 0.220	0.915 ± 0.072	0.982 ± 0.093	1.102 ± 0.133	0.929 ± 0.110
	Female	0.583 ± 0.072	0.619 ± 0.077	0.625 ± 0.047	0.687 ± 0.131	0.677 ± 0.053	0.596 ± 0.119

Adrenals	Male	0.091 ± 0.013	0.087 ± 0.010	0.084 ± 0.009	0.085 ± 0.012	0.096 ± 0.018	0.103 ± 0.011
	Female	0.100 ± 0.019	0.096 ± 0.013	0.106 ± 0.015	0.109 ± 0.017	0.097 ± 0.013	0.106 ± 0.015

Heart	Male	1.998 ± 0.208	1.947 ± 0.250	1.885 ± 0.216	1.939 ± 0.233	2.212 ± 0.173	2.087 ± 0.143
	Female	1.190 ± 0.160	1.258 ± 0.105	1.309 ± 0.140	1.255 ± 0.105	1.324 ± 0.142	1.346 ± 0.131

Thymus	Male	0.465 ± 0.070	0.472 ± 0.107	0.482 ± 0.095	0.436 ± 0.085	0.570 ± 0.051	0.476 ± 0.050^∗^
	Female	0.373 ± 0.068	0.402 ± 0.079	0.347 ± 0.036	0.387 ± 0.062	0.409 ± 0.122	0.385 ± 0.052

Brain	Male	2.358 ± 0.124	2.372 ± 0.189	2.287 ± 0.172	2.284 ± 0.167	2.487 ± 0.217	2.277 ± 0.117
	Female	2.004 ± 0.115	2.088 ± 0.162	2.032 ± 0.206	2.036 ± 0.163	2.156 ± 0.075	2.182 ± 0.105

Testes	Male	3.937 ± 0.231	3.818 ± 0.364	3.855 ± 0.253	3.833 ± 0.310	3.994 ± 0.304	4.181 ± 0.332

Ovaries	Female	0.237 ± 0.032	0.238 ± 0.030	0.250 ± 0.039	0.240 ± 0.026	0.250 ± 0.038	0.217 ± 0.010

Epididymides	Male	1.627 ± 0.216	1.797 ± 0.180	1.740 ± 0.152	1.729 ± 0.271	1.565 ± 0.378	1.696 ± 0.232

Uterus	Female	0.873 ± 0.132	0.799 ± 0.119	0.820 ± 0.148	0.865 ± 0.089	0.760 ± 0.124	0.638 ± 0.048

Thyroid with parathyroid	Male	0.039 ± 0.004	0.038 ± 0.005	0.038 ± 0.005	0.039 ± 0.006	0.032 ± 0.007	0.034 ± 0.005
	Female	0.040 ± 0.006	0.039 ± 0.005	0.039 ± 0.003	0.041 ± 0.006	0.036 ± 0.006	0.037 ± 0.006

Pituitary gland	Male	0.026 ± 0.004	0.022 ± 0.003	0.025 ± 0.003	0.023 ± 0.002	0.028 ± 0.005	0.028 ± 0.002
	Female	0.025 ± 0.004	0.025 ± 0.004	0.024 ± 0.004	0.025 ± 0.004	0.027 ± 0.004	0.032 ± 0.002^∗^

Prostate + seminal vesicles with coagulating glands	Male	4.879 ± 0.615	4.620 ± 0.683	4.529 ± 0.658	4.450 ± 0.770	5.405 ± 0.268	5.153 ± 0.695

*Note:* Values are presented as mean ± SD for main (*n* = 10) treated for 90 days and recovery (*n* = 5) groups.

Abbreviations: G, group; R, recovery.

^∗^Statistically significant change at *p* < 0.05 when compared with the respective vehicle control and recovery vehicle control group.

**TABLE 6:** Relative organ weights (%) following repeated oral administration of MUVZ to Sprague Dawley rats for 90 days.

Organ	Sex	Group					
		G1	G2	G3	G4	G1R	G4R
		Vehicle control	MUVZ	MUVZ	MUVZ	Recovery control (vehicle)	Recovery MUVZ
			1000 mg.kg^−1^ b.wt.	2000 mg.kg^−1^ b.wt.	3000 mg.kg^−1^ b.wt.		3000 mg.kg^−1^ b.wt.
Liver	Male	3.442 ± 0.354	3.524 ± 0.495	3.531 ± 0.380	3.684 ± 0.386	3.241 ± 0.348	3.409 ± 0.275
	Female	3.537 ± 0.332	3.769 ± 0.537	3.664 ± 0.367	4.108 ± 0.547^∗^	2.928 ± 0.199	3.479 ± 0.327

Kidneys	Male	0.831 ± 0.063	0.727 ± 0.069^∗^	0.744 ± 0.048^∗^	0.768 ± 0.077	0.750 ± 0.081	0.745 ± 0.063
	Female	0.771 ± 0.051	0.795 ± 0.086	0.748 ± 0.068	0.850 ± 0.084	0.704 ± 0.038	0.724 ± 0.062

Spleen	Male	0.175 ± 0.022	0.172 ± 0.037	0.155 ± 0.016	0.166 ± 0.013	0.169 ± 0.022	0.142 ± 0.012^∗^
	Female	0.198 ± 0.028	0.208 ± 0.019	0.210 ± 0.022	0.232 ± 0.052	0.195 ± 0.014	0.170 ± 0.040

Adrenals	Male	0.015 ± 0.002	0.015 ± 0.002	0.014 ± 0.002	0.014 ± 0.002	0.015 ± 0.003	0.016 ± 0.002
	Female	0.034 ± 0.006	0.032 ± 0.004	0.036 ± 0.004	0.037 ± 0.007	0.028 ± 0.003	0.030 ± 0.004

Heart	Male	0.335 ± 0.035	0.330 ± 0.037	0.320 ± 0.035	0.328 ± 0.036	0.339 ± 0.022	0.319 ± 0.020
	Female	0.402 ± 0.038	0.423 ± 0.030	0.439 ± 0.043	0.423 ± 0.038	0.381 ± 0.035	0.383 ± 0.048

Thymus	Male	0.078 ± 0.013	0.081 ± 0.019	0.081 ± 0.014	0.074 ± 0.013	0.087 ± 0.008	0.073 ± 0.004^∗^
	Female	0.126 ± 0.024	0.135 ± 0.027	0.117 ± 0.015	0.129 ± 0.015	0.117 ± 0.032	0.110 ± 0.017

Brain	Male	0.395 ± 0.024	0.403 ± 0.021	0.389 ± 0.046	0.387 ± 0.019	0.382 ± 0.033	0.349 ± 0.035
	Female	0.680 ± 0.062	0.701 ± 0.041	0.683 ± 0.079	0.686 ± 0.052	0.622 ± 0.017	0.620 ± 0.023

Testes	Male	0.660 ± 0.040	0.650 ± 0.073	0.656 ± 0.074	0.650 ± 0.047	0.613 ± 0.049	0.639 ± 0.056

Ovaries	Female	0.081 ± 0.014	0.080 ± 0.009	0.084 ± 0.014	0.081 ± 0.012	0.072 ± 0.011	0.062 ± 0.001

Epididymides	Male	0.274 ± 0.044	0.305 ± 0.027	0.296 ± 0.036	0.292 ± 0.034	0.240 ± 0.059	0.259 ± 0.035

Uterus	Female	0.297 ± 0.057	0.269 ± 0.044	0.274 ± 0.043	0.292 ± 0.039	0.219 ± 0.036	0.182 ± 0.020

Thyroid with parathyroid	Male	0.007 ± 0.001	0.006 ± 0.001	0.006 ± 0.001	0.007 ± 0.001	0.005 ± 0.001	0.005 ± 0.000
	Female	0.013 ± 0.002	0.013 ± 0.002	0.013 ± 0.001	0.014 ± 0.003	0.011 ± 0.002	0.010 ± 0.002

Pituitary gland	Male	0.004 ± 0.001	0.004 ± 0.000	0.004 ± 0.001	0.004 ± 0.001	0.004 ± 0.001	0.004 ± 0.000
	Female	0.009 ± 0.002	0.008 ± 0.002	0.008 ± 0.001	0.008 ± 0.001	0.008 ± 0.001	0.009 ± 0.000^∗^

Prostate + seminal vesicles with coagulating glands	Male	0.821 ± 0.124	0.787 ± 0.125	0.774 ± 0.156	0.756 ± 0.142	0.831 ± 0.068	0.788 ± 0.112

*Note:* Values are presented as mean ± SD for main (*n* = 10) treated for 90 days and recovery (*n* = 5) groups. Keys: mg, milligram; kg, kilogram; b.wt, body weight.

Abbreviations: G, group; R, recovery.

^∗^Statistically significant change at *p* < 0.05 when compared with the respective vehicle control and recovery vehicle control group.

### 3.7. HP

Microscopically, no test article–related changes were observed in male or female rats at the highest dose of G4 and, in comparison, with G1. However, the following microscopic changes were observed in the vehicle control and G4, i.e., perivascular infiltration of mononuclear cells (liver), perivascular chronic inflammation and perivascular infiltration of mononuclear cells (lungs), vacuolation of zona fasciculata (adrenal glands), vacuolation of zona fasciculata (adrenal glands), atrophy/degeneration atrophy in seminiferous tubules (testes) and perivascular infiltration of mononuclear cells (prostate) and unilateral follicular cyst (ovaries).

The lesions observed in vehicle control and high‐dose MUVZ animals are common background findings in rats. Hence, these were considered toxicologically irrelevant and unrelated to the test item. No significant changes were observed during the oestrous cycle examination and were further confirmed by the histology of the respective target reproductive organs (Figure 1).

**FIGURE 1:**
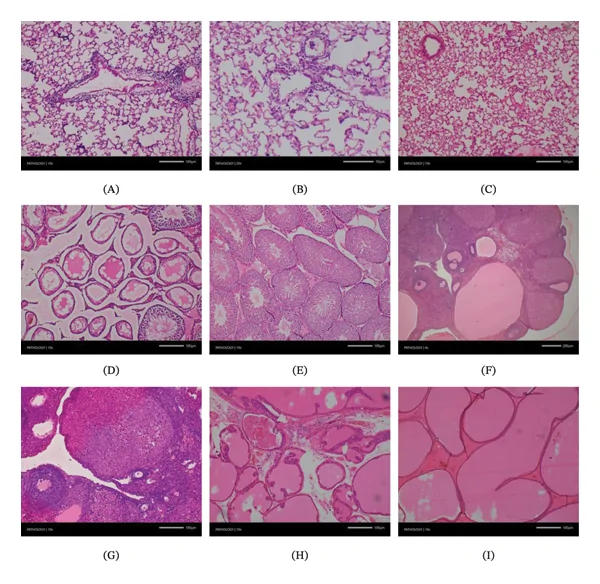
Histopathological representation (10x). (A) G1‐001: lungs—inflammation, chronic, perivascular; (B) G1‐012: lungs—infiltration, mononuclear cells, perivascular; (C) G1‐002: lungs—normal; (D) G4‐065: testes—degeneration/atrophy, seminiferous tubules; (E) G1‐003: testes—normal; (F) G1‐015: ovary—cyst, follicular; (G) G1‐010: ovary—normal; (H) G1‐008: prostate—infiltration, mononuclear cells, perivascular; (I) G1‐002: prostate—normal.

## 4. Discussion

Subchronic oral toxicity of MUVZ was screened in both sexes of Sprague Dawley rats. No MUVZ‐related changes were evidenced in clinical signs, b.wt, feed consumption, clinical pathology and histopathological evaluation across G1–G4 and G1R–G4R groups of both sexes during 90 days of exposure. Statistically significant changes in serum TSH (G3, G4 and G4R of both females and males) and T3 (G4 in males and females and G4R males) concentrations were observed. However, these changes were not accompanied by corresponding alterations in serum T4 concentrations, absolute or relative thyroid weights or gross and microscopic pathological findings in the thyroid gland. Furthermore, the observed hormonal changes were inconsistent in direction, with increases observed in the main study G3 and G4 and decreases in G4R recovery groups, and were not associated with any dose‐related pathological changes. Since these findings lacked corroborative evidence of thyroid toxicity, they were considered incidental, nonadverse and unrelated to MUVZ administration.

Based on these findings, the NOAEL of MUVZ was found to be 3000 mg.kg^−1^ b.wt.day^−1^. For the human equivalent dose (NOAEL/6.2 [conversion factor for rat]), the dose in rats could be equated to 484 mg.kg^−1^ b.wt. Considering an average human b.wt (60 kg), the average daily intake of MUVZ was 29 mg.day^−1^, suggesting a margin of safety versus the NOAEL in rats, resulting from the highest dose tested and feasible for testing.

## 5. Conclusion

Based on the findings of a 90‐day subchronic study, the novel proprietary blend of MUVZ has been found to be nontoxic and safe at tested doses. Hence, it is concluded that the NOAEL MUVZ is 3000 mg.kg^−1^ b.wt. Day [1] in rats.

## Funding

This study was funded by Enovate Biolife, Mumbai, India.

## Disclosure

The author accepted responsibility for the entire content of this manuscript and approved the submission.

## Conflicts of Interest

The author declares no conflicts of interest.

## Supporting Information

Additional supporting information can be found online in the Supporting Information section.

## Supporting information


**Supporting Information** **Supplementary Figure S1a**: By the HPLC method, negundoside (*Vitex negundo*) in MUVZ was identified using the Inertsil ODS C18 column, with Mobile Phase A (potassium dihydrogen phosphate + *ortho*‐phthalaldehyde) and Mobile Phase B (acetonitrile) at a flow rate of 0.8 mL/min. **Supplementary Figure S1b:** The retention time of negundoside in MUVZ was observed at 23 min, matching the peak obtained from the reference negundoside standard solution. **Supplementary Figure S2a:** By the HPLC method, for gingerols (*Zingiber officinale*), a Hypersil BDS C18 column was used with Mobile Phase A (acetonitrile: orthophosphoric acid in water) and Mobile Phase B (acetonitrile) at a flow rate of 1 mL/min. **Supporting Figure S2b:** Relative retention time of 6‐gingerol, 8‐gingerol and 10‐gingerol in MUVZ was found to be 0.8, 1.5 and 3.4 min, respectively. **Supporting Figure S3a:** By the HPLC method, for total gingerols (*Zingiber officinale*), a Hypersil BDS C18 column was used with Mobile Phase A (acetonitrile: orthophosphoric acid in water: methanol) and Mobile Phase B (acetonitrile) at a flow rate of 1 mL/min. **Supporting Figure S3b:** Gingerol content was found to be 3%‐4%. **Supporting Figure S4:** MUVZ has been subjected to estimation of total flavonoid content (should not be less than 15%) as rutin hydrate equivalent by the UV spectrophotometer.

## Data Availability

The data that support the findings of this study are available from the corresponding author upon reasonable request.
