# Supplementary material for: 90‐Day Repeated‐Dose Toxicological Assessment of MUVZ in Sprague Dawley Rats: Subchronic Study
Source: J Toxicol. 2026 Oct 13;2026:9540593. doi: 10.1155/jt/9540593 (PMC13659643; doi:10.1155/jt/9540593)
Supplement: Supplementary file 1 [file JT-2026-9540593-s001.docx]

**Supplementary Figure S1a**. High-Performance Thin-Layer Chromatography conditions of *Vitex Negundo (Negundoside)* in MUVZ™. Inertsil ODS C18 column was used with Mobile Phase A (Potassium dihydrogen phosphate + ortho-phthalaldehyde) and Mobile Phase B (Acetonitrile) at a flow rate of 0.8 ml/min

**Supplementary Figure S1b**. High-Performance Thin-Layer Chromatogram of *Vitex Negundo (Negundoside)* in MUVZ™. Retention time (RT) of Negundoside was observed at 23 minutes, matching the peak with the reference standard solution.

**Supplementary Figure S2a**. High-Performance Thin-Layer Chromatography Conditions of *Zingiber Officinale (Gingerols)* in MUVZ™. Hypersil BDS C18 column was used with Mobile Phase A (Acetonitrile: Ortho-phosphoric acid in water) and Mobile Phase B (Acetonitrile) at a flow rate of 1 ml/min.

**Supplementary Figure S2b**. High-Performance Thin-Layer Chromatograms of *Zingiber Officinale (Gingerols)* in MUVZ™. Relative Retention time of 6-Gingerol, 8-Gingerol, and 10-Gingerol in MUVZ™ was found to be 0.8, 1.5 & 3.4 minutes, respectively

**Supplementary Figure S3a**. High-Performance Thin-Layer Chromatography Conditions and chromatogram of *Total Gingerols* in MUVZ™. Hypersil BDS C18 column was used with Mobile Phase A (Acetonitrile: Ortho-phosphoric acid in water: Methanol) and Mobile Phase B (Acetonitrile) at a flow rate of 1 ml/min

**Supplementary Figure S3b**. High-Performance Thin-Layer chromatogram of *Total Gingerols* in MUVZ™. Gingerol content was found to be 3-4 %.

**Supplementary Figure S4**. UV spectrometer Conditions and observations of *Total flavonoids as Rutin Hydrate equivalent* in MUVZ™. Total flavonoid content should not be less than 15%.
